# EPG analysis of stylet penetration preference of woolly apple aphid on different parts of apple trees

**DOI:** 10.1371/journal.pone.0256641

**Published:** 2021-08-24

**Authors:** Hao Zhou, Xiumei Tan, Ziwen Teng, Lingjun Du, Hongxu Zhou

**Affiliations:** Key Laboratory of Integrated Plant Disease and Pest Control, China-Australia Joint Institute of Agricultural and Environmental Health, College of Botanical Medicine, Qingdao Agricultural University, Qingdao, China; University of Carthage, TUNISIA

## Abstract

Woolly apple aphid (WAA), *Eriosoma lanigerum* (Hausmann), is an important global pest that feeds on *Malus* species. We studied the feeding preference of WAA on apple trees in the field for two consecutive years and in the laboratory we used electronic penetration graphs (EPG) to record the stylet penetration behavior of WAA on different parts of apple trees. We found that in the field WAA fed primarily on twigs and branches, not on leaves and fruits. Six EPG waveforms were produced during WAA probing on shoots, trunks and leaves of apple trees, including the non-penetration wave (np), the stylet pathway phase wave (C), the intracellular feeding wave (pd), the xylem feeding wave (G), waves indicative of release of saliva into the phloem (E1), and a wave indicative of ingestion from phloem (E2). In the laboratory, aphids only successfully fed on shoots, trunks and leaves, not on fruits. The EPG parameters on the phloem of shoots were significantly higher than those on trunks, indicating WAA prefer to feed on shoots. These laboratory findings explain the relative field feeding preference of WAA on different parts of apple trees, which occurs primarily on branches, barks, and young twigs in orchards, especially on young twigs.

## 1 Introduction

Aphids feed on a wide range of plant tissues, including leaves, young shoots, young twigs, branches, flowers, roots, and fruits [1]. Aphids that feed on trees account for 40% of aphid species worldwide [2]. Arboreal aphids, such as *Myzus persicae* (Sulzer) or *Aphis citricola* van der Goot, usually feed on leaves or leaf petioles, cause leaves to become chlorotic, and may lead to some defoliation, but rarely cause tree death [3, 4]. While some aphids, such as woolly apple aphid (WAA) *Eriosoma lanigerum* (Hausmann), feed mainly on the trunk, branches and roots, even causing the tree death [2, 5].

WAA is one of the world’s most important invasive apple pests, originating from eastern North America but now found in most apple-growing areas of the world [6]. This aphid species infests the crown and root systems of apple trees, but does not feed on the leaves [7]. However, it causes more damage to the host plant than do aphids that feed on leaves. The damaged tissues, stimulated by the salivary gland secretions of the WAA, form abnormal growths or galls that affect the transport of water and nutrients within the plant, weakening the tree [8, 9]. The wounds caused by the frequent rupturing of the galls promote aphid feeding and the invasion of plant pathogens [10, 11]. WAA injure twigs and branches, but rarely directly damage leaves or fruits. Understanding the reasons for the feeding niche of this species is of significance for the management of WAA.

The technique known as electrical penetration graphing (EPG) can detect differences in the feeding mechanism of WAA when they feed on apple varieties with different levels of resistance. EPG has the advantages of being a rapid method to make behavioral measurements, as well as having high sensitivity and convenience [12–14]. The greatest advantage of EPG lies in its ability to accurately record the starting and ending times of the stylet penetration activities and the frequency of each part of the probing pathway of the insect’s stylet in plant tissues, including penetration of the epidermis, salivation when the stylet reaches the phloem, passive phloem ingestion, and xylem sap ingestion [15].

Some of these behavioral feeding parameters have been shown to reflect the effects of various physical and chemical factors in plant tissues that can affect penetration and feeding [16].

To clarify the patterns of WAA feeding in the field on different parts of apple trees, we observed the level of infestation and damage from WAA on 15 apple cultivars on different parts of apple trees for two consecutive years. In addition, we used EPG technology in the laboratory to observe differences in the insertion of the stylet and probing behavior of WAA in different parts (shoots, trunks, leaves and fruits) of the apple tree on one cultivar (Red Delicious Starkrimson). We then analyzed the differences in the types of waveforms associated with probing activities and feeding of WAA, when aphids were placed on different parts of apple trees of Starkrimson in relation to specific feeding behaviors.

## 2 Materials and methods

### 2.1 Field studies on the effect of plant part on WAA feeding

#### (a) Effect of plant parts

Apple orchard number 1 (2001 x 1334 m) was dominated by Red Fuji (tree-age: 15–20 year), and was located in Laiyang, Shandong Province, China (120.71E, 36.97N). At this site, our objective was to compare the abundance of WAA on a single cultivar on different parts (twigs, branches, leaves and fruits) of the apple trees. The orchard was treated with chemical pesticides at the peak of pest occurrence (late May to late July), pesticides used and timings were stated in our previous study [17]. The survey was conducted every 5–7 days from 2008 to 2009, and the samplings were carried out as described below in part (c).

#### (b) Effect of apple cultivar

At the second field site, located on the experiment land of College of Horticulture, Qingdao Agricultural University (Qingdao, Shandong Province, China) (120.33E, 36.07N), we observed WAA populations in an orchard (2001 x 667 m) containing three-year-old 15 apple cultivars: Bedan, Ben Davis, Petite Jaune, Genera Early, White Winter Pearmain, Spartan, Kermerrien, Marie Menard, Jonathan, Douce Moen, Pink lady, Golden Delicious, Dabinette, Douce Coetligne, and Juliana. No chemical control treatments were applied for WAA in this orchard.

#### (c) Description of sampling methods

For experiment *(a)* we used a 5-point random sampling method in which one tree was selected at each of 5 locations within the orchard (tree height being basically the same), and 3 main branches about two-meter long were selected for the investigation of WAA on leaves, fruits, twigs (thin and lignified stem growing within one year) and branches (old branches growing for more than one year); for experiment *(b)* we chose one sample tree randomly for each cultivar and calculate WAA numbers and colony size on different parts of the whole tree. Investigation method for population size of WAA was referred to our previous paper [18].

### 2.2 EPG observations on aphid feeding behavior

#### 2.2.1 Plants and insects

One-year-old seedlings of apple trees (cultivar Starkrimson) of similar size were collected from the Shandong Yantai nursery (YanTai, Shandong, China) (121.39E,37.52N), transplanted into plastic pots (25 x 25 x 25 cm, pots provider: Taizhou Zhengda Horticulture Company), and then held in a greenhouse (25 ± 3°C, natural light, 40–45% RH) until they reached 60–70 cm in height. Apple fruits used in the experiments were Starkrimson apples of similar size and color, obtained from an orchard using normal local management practices. The fruits were washed with water before their use in the experiment to remove contaminants from fruit surface.

WAAs to be used in the experiments were obtained from a colony reared in a greenhouse Center for Advanced Invertebrate Cell Culture and Cell Engineering of Qingdao Agricultural University (QingDao, Shandong, China) (120.33E, 36.07N). This colony was reared for several generations in a greenhouse (25 ± 3°C, natural light, 40–45% RH) on 2-year-old apple trees taken from a non-pesticide-treated orchard. After three generations of such rearing, adult aphids (apterous females) of the same age and size were used for EPG studies. Aphids were only used once.

#### 2.2.2 EPG analysis

Adult aphids used in the experiments were collected from the colony to a clean Petri dish, using a fine brush. The aphids were checked microscopically to ensure that the mouthparts were not damaged. The white wax wool secreted from their bodies was removed using distilled water and a fine brush (Prevent white wax wool from interfering with electrical signal). All experiments were performed at ambient temperature (25 ± 2°C), 14:10 L:D photoperiod, and 60–70% RH and the EPGs of aphids were recorded using a Giga-8 DD-EPG system (Input voltage = 220V; Giga-8 DD; EPG Systems, Wageningen, the Netherlands). In order to shield electromagnetic interference from the outside environment, experiments were conducted in Faraday cages (80 x 60 x 100 cm). The insect-linked and plant-linked electrodes were attached to a biological current amplifier. The gold wire electrode was 3–4 cm long and 18 μm in diameter. The terminal of the insect-electrode lead was attached to the pronotum of an aphid using a water-soluble conductive silver paste, and the plant-electrode was inserted in the soil around the apple seedling or, in the test with fruit, was inserted directly into the fruit. Wired aphids were placed in a clean Petri dish for 1 h to recover from the stress of installment of the wire tether. The aphids were then placed individually on shoots (new part of growing tree which is unlignified), the undersides of leaves, on trunks (the main stem of apple seedlings), or skin of the fruit for testing. When the stylets of an aphid pierced the plant’s tissue, the circuit was closed and amplified, and then transformed into a digital signal, which was stored in the computer by amplification via Giga-8d control unit. This digital signal was then transformed into a waveform and displayed on the screen using the Stylet+d software, which was also used to interpret and convert the waveforms into digital files ready for pattern recognition. We used the Stylet+a software to distinguish and manually annotate the waveform events according to Tjallingii and Tjallingii and Hogen [19, 20], and we obtained a data file including the waveform codes.

For each part of the apple tree, the stylet penetration activities were recorded for 15 aphids per treatment over an 8 h time period for each aphid individual. Aphids that did not show phloem ingestion or did not complete the phloem phase during the 8 h recording period were not included in the data.

### 2.3 Data analysis

Statistical analyses of field observations were performed in SPSS v.19.0 (SPSS, Chicago, IL, USA). Data on number of aphids were analyzed with one-way ANOVA, followed by the Least Significant Difference test (LSD) for multiple comparisons.

Statistical analyses of EPG data for aphid feeding behavior bouts, among different parts of the apple tree were performed in SPSS v.19.0 (SPSS, Chicago, IL, USA). Data on duration and counts of events were analyzed with one-way ANOVA, followed by the use of Least Significant Difference test (LSD) for multiple comparisons among the various EPG response variables.

## 3 Results

### 3.1 Field seasonal trends of WAA on different parts of apple tree

**Fig 1:**
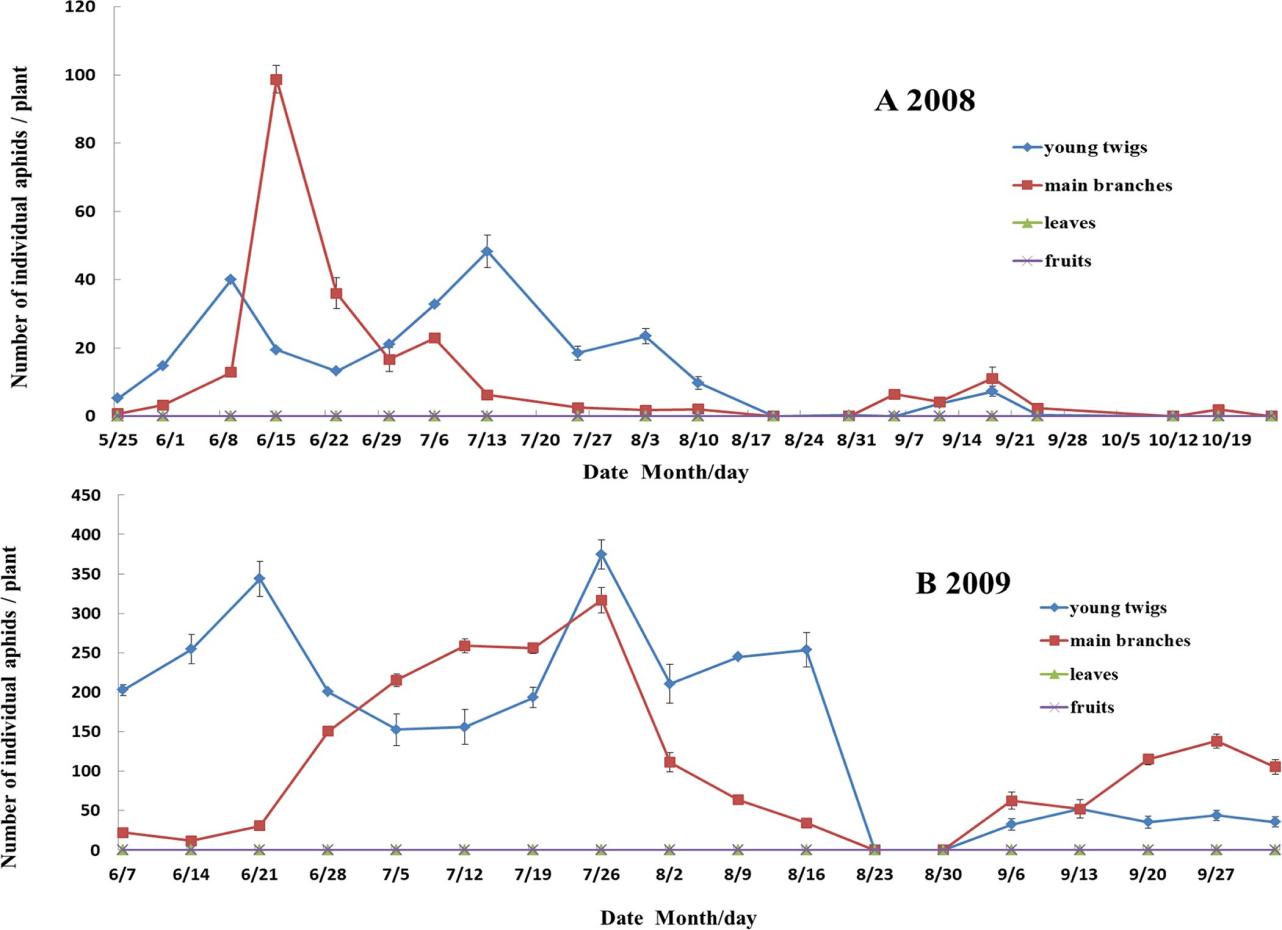
Dynamics of woolly apple aphid populations on different parts of apples, in an apple orchard in Laiyang, Shandong Province, China, in (A) 2008 and (B) 2009.

Trends of WAA density on one apple cultivar (Red Fuji) in 2008 and 2009 revealed that WAA preferred to feed on young twigs and branches, no WAA was found on leaves and fruits (Fig 1). In mid-June 2008, the number of aphids on main branches (98.7 aphids / plant) was significantly higher than that on young twigs (19.5 aphids/ plant) (*P<0*.*05*). In 2009, we found the same pattern as in 2008. From late June to mid-July 2009, the number of apple aphids on main sample branches (260.8 aphids / plant) was significantly higher than that on young twigs (156 aphids / plant) (P<0.05).

### 3.2 Effect of 15 apple cultivars on WAA density by plant part

**Fig 2:**
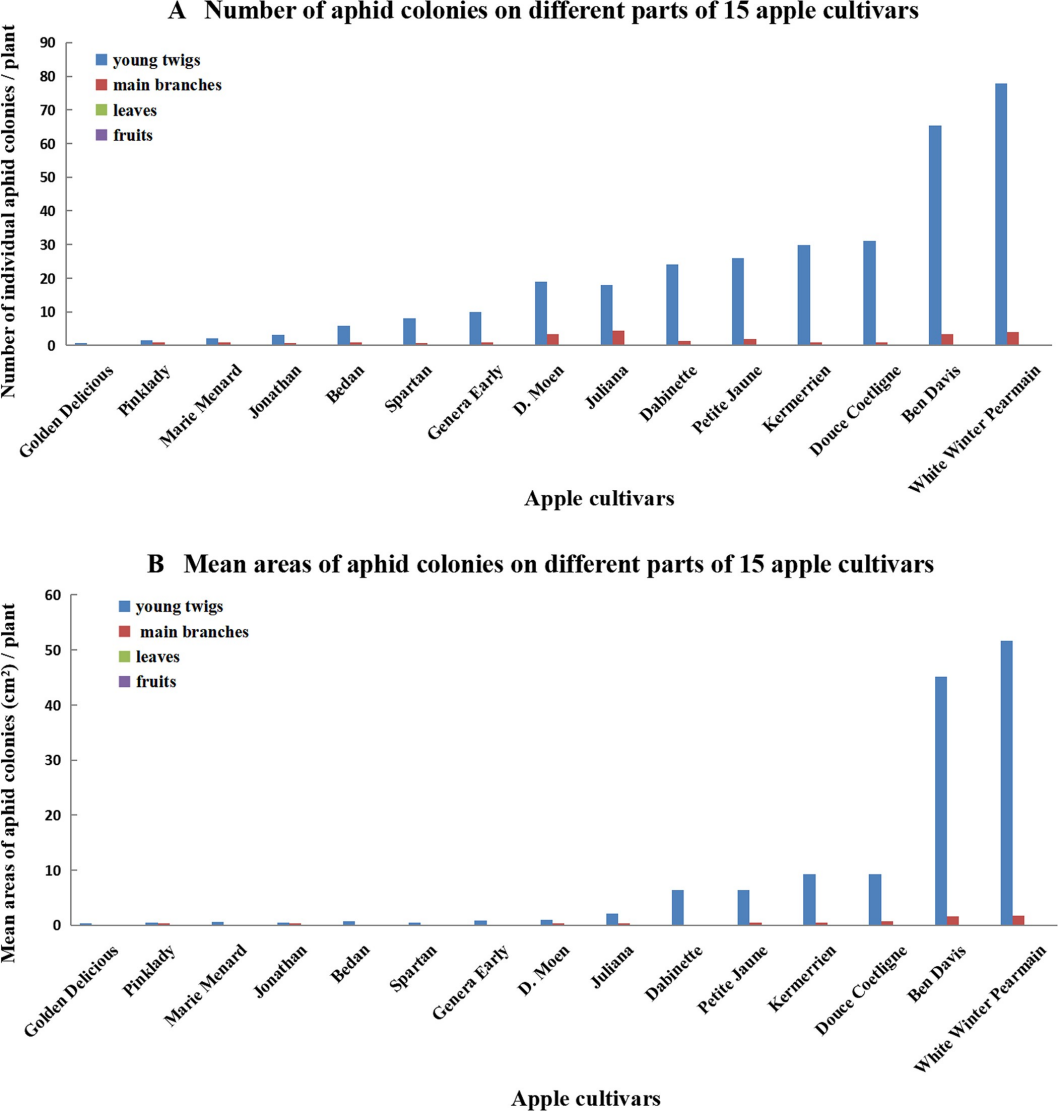
(A) Numbers and (B) area (cm^2^) of aphid colonies per part on various plant parts on 15 apple varieties, in an apple orchard in Qingdao, Shandong Province, China, in June 20, 2010.

For all 15 apple cultivars, both aphid number (Fig 2A) and area of aphid colonies (Fig 2B) were greater on young twigs than on main branches. No WAAs were found on leaves or fruits. On most cultivars, WAAs were found only on young twigs. These observations showed that WAA selection of feeding sites was not affected by apple cultivar.

The orchard investigation in the above 2 experiments indicated that WAA infested twigs and branches rather than leaves and fruits.

### 3.3 EPG observations of aphid feeding behavior on different parts of apple tree

The number of probes on shoots was significantly lower than that on fruits or abaxial side of the leaves, but not significantly different from that on trunks (*P* < 0.05) (Table 1). At the same time, the total duration of np (non-penetration events) on the leaves (8641.2 sec) and fruits (9110.06 sec) was significantly higher than that on shoots (296.23 sec) or trunks (3594.88 sec) (*P* < 0.05). Meanwhile, time to first C (the time when the stylet first pierced the epidermis) on the fruits (1737.31 sec) was significantly higher than that on shoots (318.15 sec), trunks (558.36 sec), or leaves (643.51 sec) (*P* < 0.05). However, the three EPG parameters of total duration of C, total duration of G (associated with xylem sap ingestion) and count of pd need to be analyzed in combination with their actual biological significance. The total duration of G on the leaves (5600.43 sec) was significantly higher than that on shoots (1643.42sec), trunks (1272.05 sec), or fruits (2419.34 sec).

**Table 1:** Comparison of EPG parameters of woolly apple aphid, *Eriosoma lanigerum*, during non-phloem feeding on four parts of apple trees (var. Starkrimson).

EPG Parameters	Plant parts (Mean ± SE)			
	shoots	trunks	leaves	fruits
number of probes	1.88 ± 1.25b	3 ± 1.51ab	3.36 ± 2.44a	3.89 ± 2.52a
Total duration of np (s)	296.23 ± 221.23b	3594.88 ± 1912.97b	8641.2 ± 6189.22a	9110.06 ± 4966.50a
Total duration of C (s)	15094.02 ± 4191.80a	11772.93 ± 5203.16ab	10387.19 ± 6320.88b	15610.13 ± 5908.79a
Total duration of G (s)	1643.42 ± 3009.74b	1272.05 ± 1375.06b	5600.43 ± 3648.44a	2419.34 ± 3197.51b
Count of pd	69.93 ± 30.65a	42.5 ± 12.62b	16.33 ± 6.73c	43.57 ± 20.33b
Time to first C(s)	318.15 ± 160.58b	558.36 ± 353.63b	643.51 ± 601.8b	1737.31 ± 855.07a

Different letters in the same row indicate significant difference at *P* <0.05 level by LSD test (Least Significant Difference test). The letter (s) equals seconds. The same below.

During aphid exploration to detect phloem and begin phloem sap ingestion, the phases we measured included (1) the % of aphids reaching phloem, (2) the total duration of E2, (3) the number of E2 events longer than 600s (When E2 was longer than 600s, it was considered that aphids continued to suck in phloem) [15], (4) the % of aphids in the first phloem phase that reach sustained phloem feeding, (5) the count of E2 events, and (6) the contribution of E2 phase to total phloem phase (indicative of the tissue preference of WAA for feeding) (Table 2). Overall, 81.3, 73.3, 46.2, and 0.0% of the aphids attained phloem feeding on shoots, trunks, leaves and fruits, respectively. The average number of E2 events per aphid on shoots (2.62 events per aphid) and trunks (1.91) was significantly higher than on leaves (0.8) or fruits (0) (*P* <0.05). At the same time, the total duration of the E2 events (10,338.31 sec) on shoots was significantly higher than on trunks (6745.16 sec) and even more significantly higher than on leaves (2535.32 sec) and fruits (0 sec) (*P* <0.05). For the parameter of E2 feeding events > 600 sec in duration, shoots (10125.38 sec) were significantly higher than trunks (6654.22 sec) and even more significantly higher than leaves (2158.78 sec) and fruits (0sec) (*P* <0.05).

**Table 2:** Comparison of EPG parameters of woolly apple aphid *Eriosoma lanigerum* during phloem feeding on four different apple parts.

EPG Parameters	Plant parts (Mean ± SE)			
	shoots	trunks	leaves	fruits
% aphids reaching phloem	81.25	73.33	46.15	0
Total duration of E1 (s)	1421.98 ± 685.91a	1104.63 ± 843.93a	70.61 ± 115.91b	0 ± 0b
Total duration of E2 (s)	10338.31 ± 5215.94a	6745.16 ± 4708.05b	2535.32 ± 3162.57c	0 ± 0b
E2>600 s	10125.38 ± 5652.02a	6654.22±4818.58b	2158.78±3039.54c	0 ± 0b
% aphids in 1st phloem phase with sustained phloem ingestion	56.25	40	38.46	0
Count of E1	4.18 ± 1.40a	2.18 ± 1.17b	1.09 ± 1.38c	0 ± 0d
Count of E2	2.62 ± 1.61a	1.91 ± 0.94a	0.80 ± 1.03b	0 ± 0b
Contribution of E1 to phloem phase (%)	13.78 ± 7.05a	11.61 ± 9.03a	9.46 ± 18.8ab	0 ± 0b
Contribution of E2 to phloem phase (%)	86.22 ± 7.04a	88.39 ± 9.03a	37.42 ± 44.77b	0 ± 0b
Time to first E1(s)	7965.98 ± 2752.79a	8216.27 ± 6135.90a	11077.81 ± 5595.14a	/
Time from first probe to first E2(s)	12248.03 ± 6005.27a	13709.36 ± 4288.7a	12464.54 ± 4474.11a	/

Different letters in the same row indicate significant difference at *P* <0.05 level by LSD test (Least Significant Difference test).

**Fig 3:**
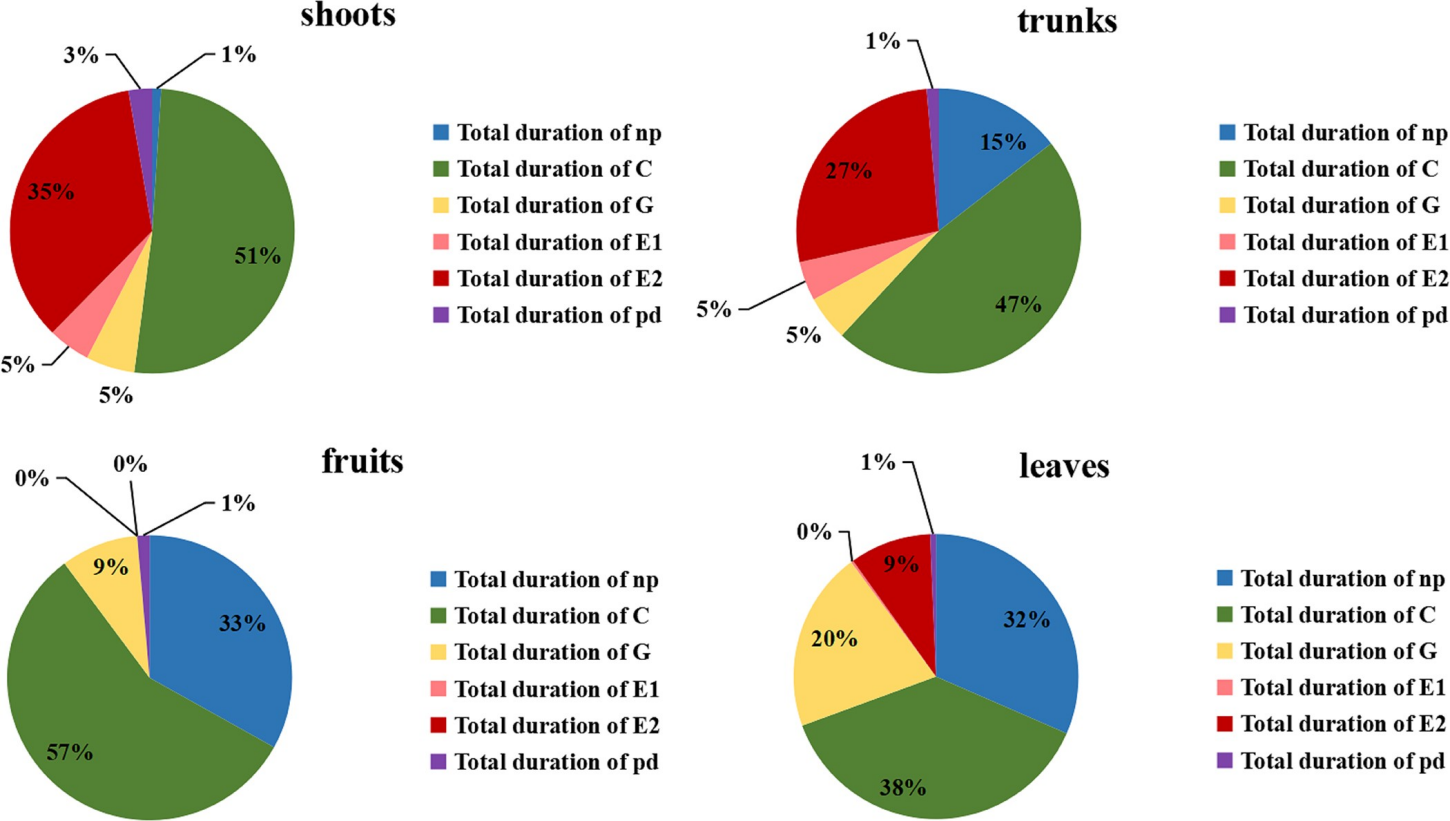
The percentage of various waveforms’ (average duration) of per aphid (*Eriosoma lanigerum*) feeding on different parts of apple tree for eight hours.

Fig 3 showed the difference in the proportions of different feeding wave patterns in aphids feeding on different parts of the apple trees. When aphids fed on shoots and trunks, the proportion of E waves (feeding on phloem) was 40 and 32%, respectively, which was higher than on leaves (9%). There were no E waves on the fruits. The proportion of np waves values on shoots (1%) were significantly lower than on trunks (15%), leaves (32%), and fruits (33%). The proportion of G waveform was longest on leaves (20%), followed by fruits (9%), while shoots (5%) and trunks (5%) were similar.

## 4 Discussion

We found that different apple cultivars did not affect the selection of feeding sites by WAA, but rather that WAA preferred to feed on young twigs, followed by main branches rather than leaves and fruits on all tested cultivars in the orchards. In order to further clarify the pattern of WAA damage to different parts of the apple tree and the behavioral mechanism determining how they choose to feed, we did EPG experiment in the laboratory. In terms of plant structure, what shoots and trunks to one-year-old seedlings are what twigs and branches to multiple-year-old apple trees in orchards, so we tested feeding responses to shoots, trunks and leaves on one-year-old cultivar and to fruits, and measured aphid feeding using the EPG process. We observed that feeding preferences seen in the field were consistent with rates, in EPG observations, of the non-phloem and phloem probing or feeding.

Phytophagous insects gradually adapt the details of their specific feeding range over the course of their co-evolution with available plant hosts [21]. Therefore, different aphids have developed different favorable feeding sites and occupy different niches to promote their survival [20]. The choice of host and feeding sites by herbivorous insects depends to a large extent on the secondary plant compounds and the nutrient content of the tissues they feed on [14].

EPG was used to record the waveforms of WAA on shoots, trunks, and leaves, and the frequencies and durations of six (np, C, pd, G, E1 and E2) waveforms were characterized. These waveforms had the same shape, amplitude and frequency of EPG signal waves observed for WAA by Sandanayaka and Hale [22] and Zhou et al. [23]. For the waveform records of WAA on fruit, three waveforms (C wave, Pd wave, and G wave) were found, but there were no E1 or E2 waves, which are indicative of phloem feeding. These results show that WAA does not feed on fruit phloem, which may be related to the structure or nutritional components of fruit.

Host selection by aphids uses various sensory and behavioral mechanisms to locate and identify potential host plants and plant parts [24]. Initially, aphids can detect physical and chemical factors on the surfaces of plant tissues before penetrating the epidermis of the plant [25]. In terms of EPG parameters, the total length of np waves in apple fruits and leaves (33% and 32% of the total time), was significantly higher than that on young shoots (1% of the total time) or trunks (15% of the total time). In addition, the time to first probing was tender shoots < trunks < leaves < fruits. From the above parameters, we can infer that chemical cues, visual and contact cues factors on the surface of apple fruits and leaves do little to stimulate WAA probing.

At the same time, the waxy substance of fruit and leaf epidermis, the texture of the epidermis and the cell composition may be not suitable for WAA sucking. Ge et al. [26] found that the stylet and beak of WAA lack external chemoreceptors. However, during the process of stylet penetration, plant sap may be ingested and come into contact with the taste organs of the pharynx, which could affect aphids’ preference for plants [27]. The C wave is the waveform of the aphid’s probing process in the mesophyll cell space. Therefore, during the normal probing process, the length of the C wave also indicates the aphid’s preference for hosts. The total time of the C wave on shoots and trunks was longer than that on leaves, which also indicates that WAA prefer to feed on shoots and trunks. Moreover, the number of probes on leaves and fruits was significantly higher, which strongly supports the assumption that there were external factors of the epidermis and tissues of leaves and fruits that interrupted probing behavior.

This study also found that the total duration on leaves of the G wave (20% of the total experimental time) was significantly higher than on fruits (9%), shoots (5%), or trunks (5%). G waves are principally indicative of water-feeding in xylem. The G waveform appears in most phloem sap sucking insects [28]. Therefore, we speculated that the nutrient concentration in the leaves may not be suitable for WAA feeding, even though water from leaf xylem is acceptable. Or perhaps it may be physical or chemical factors in the leaves that interfere with phloem-feeding by WAA.

Plant nutrients and toxic (total phenols, protease inhibitors, lectins, etc.) substances can directly affect the suitability of plants for use as hosts by herbivorous insects [29]. Especially for aphids, the phloem quality can affect aphid feeding behavior [30]. Phloem sap contains amino acids, useful as a nitrogen source, and sugar, a carbon source. Therefore, a final factor in the chain of stimuli influencing host acceptance by aphids may be the ratio of sugar and amino acids in the phloem, and the exact composition of amino acids and their concentration in phloem [31–33]. In addition, the secondary plant metabolites contained in phloem sap can also affect aphid feeding [34–36]. We found that aphids strongly preferred to feed on phloem of young shoots and trunks, rather than on leaves or fruits. Total duration of E2, count of E2, contribution of E2 to phloem phase (%), and total duration of E2 > 600s are all parameters related to phloem feeding, for which the values for twigs and branches were significantly higher than for leaves. Among them, the parameters total duration of E2 and total duration of E2 > 600s of shoots were significantly higher than those for trunks. According to the time of feeding on phloem, the time of feeding on shoots (40% of total time) was higher than that on trunks (32%). At the same time, aphids in the first phloem phase demonstrated sustained phloem ingestion shoots, trunks, and leaves, being 56.3, 40.0, and 38.5%. There was no phloem feeding on fruits. According to the above analysis, we can see that WAA’ preference among parts of apples (for phloem feeding) was shoots > trunks > leaves > fruits, which is consistent with the preferences of WAA feeding in the field.

The physical and chemical components of the epidermis, and the nutrition and resistant substances of phloem are different between plants and within plants, which affect the feeding and reproduction behavior of aphids [30]. Therefore, EPG parameters measured for phloem and non-phloem feeding suggest that as apple shoots grow and become twigs, which gradually become more lignified, nutrients in the phloem and the metabolites that attract aphids gradually decrease. The field investigation also shows that at low infestations, the aphid is confined to the trunk and large branches but disperses to establish colonies on twigs or new lateral growths during peak populations, WAA prefer to feed on young twigs [13, 30, 36]. The fact that some WAA can insert stylets in apple leaves suggest that leaves contain some nutrients, but their concentration may not be high enough to sustain prolonged WAA feeding, or conversely, physical or chemical factors in leaves may interfere with WAA feeding. Future studies should examine the physical and chemical composition of different parts of apples to better inform our understanding of the preference of WAAs to various parts of their host.
